# Crtc1 Deficiency Causes Obesity Potentially via Regulating PPARγ Pathway in White Adipose

**DOI:** 10.3389/fcell.2021.602529

**Published:** 2021-04-12

**Authors:** Yimeng Hu, Jian Lv, Yu Fang, Qiang Luo, Yuan He, Lili Li, Mingxia Fan, Zhihua Wang

**Affiliations:** ^1^Department of Endocrinology, Renmin Hospital of Wuhan University, Wuhan, China; ^2^Department of Cardiology, Renmin Hospital of Wuhan University, Wuhan, China; ^3^Central Laboratory, Renmin Hospital of Wuhan University, Wuhan, China; ^4^Animal Center, Renmin Hospital of Wuhan University, Wuhan, China; ^5^Shenzhen Key Laboratory of Cardiovascular Disease, Fuwai Hospital Chinese Academy of Medical Sciences, Shenzhen, China

**Keywords:** lipid metabolism, adipose, obesity, Crtc1, Pparγ (peroxisome proliferator-activated receptor gamma)

## Abstract

Obesity is characterized by excessive fat accumulation and associated with glucose and lipid metabolism disorders. Crtc1, a transcription cofactor regulating CREB activity, has been involved in the pathogenesis of metabolic syndrome; however, the underlying mechanism remains under debate. Here we generated a Crtc1^–/–^ mouse line using the CRISPR/Cas9 system. Under normal feeding conditions, Crtc1^–/–^ mice exhibited an obese phenotype resultant from the abnormal expansion of the white adipocytes. The development of obesity in Crtc1^–/–^ mice is independent of alterations in food intake or energy expenditure. Moreover, Crtc1^–/–^ mice were more prone to insulin resistance and dyslipidemia, as evidenced by higher levels of plasma glucose, insulin and FABP4 than wildtype mice. Transcriptome analysis in liver and epididymal white adipose tissue (eWAT) showed that the fat accumulation caused by Crtc1 deletion was mainly related to lipid metabolism in adipose tissue, but not in liver. GSEA and KEGG analysis identified PPAR pathway to be of the highest impact on lipid metabolism in eWAT. This regulation was independent of a direct interaction between CRTC1 and PPARγ. Our findings demonstrate a crucial role of Crtc1 in regulating lipid metabolism in adipose during development, and provide novel insights into obesity prevention and therapeutics.

## Introduction

Obesity is associated with risks of numerous co-morbidities including insulin resistance, dyslipidaemia, hypertension, cardiovascular disease and cancer (Emerging Risk Factors et al., 2011; Seravalle and Grassi, 2017; Malden et al., 2020). During the development of obesity, expansion of white adipose tissue (WAT) may be derived from an increase in the size (hypertrophy) and/or in the number of adipocytes (hyperplasia), causing compromised capacity of WAT to sense nutrients and further result in ectopic lipid deposition (Jo et al., 2009; Sun et al., 2011). Functionally, subcutaneous white adipose tissue (sWAT) has greater adipogenic differentiation ability than epididymal white adipose tissue (eWAT) in mice (Carobbio et al., 2017). However, eWAT is a major tissue used when sWAT cannot accommodate excess fat deposition, and fat accumulation in eWAT mirrors metabolic risk to a certain degree (Smith, 2015). Many etiological factors, including age, gender, sex hormones, genetics, ethnicity, lifestyle, and etc., contribute to fat accumulation (Tchernof and Despres, 2013). How to precisely restrict the development of obesity in patients with variable genetic backgrounds remains a major challenge in this field.

CREB regulated transcription coactivator 1 (Crtc1), a member of the family of CREB transcription co-factors, functions as a transducer to regulate CREB activity in the absence of cAMP stimulus (Conkright et al., 2003; Iourgenko et al., 2003). Crtc1^–/–^ mice exhibit hyperphagia, obesity, infertility and cardiac hypertrophy (Altarejos et al., 2008; Morhenn et al., 2019). Previous studies have reported Crtc1 mediates the central effects of leptin on satiety and reproduction (Altarejos et al., 2008), and participates in the regulation of circadian clock (Jagannath et al., 2013), peripheral glucose metabolism (Kim et al., 2015), cardiac function and growth (Morhenn et al., 2019), and memory formation (Uchida et al., 2017). Reduced hypothalamic expression of anorexigenic neuropeptide genes is one of the reasons that Crtc1^–/–^ mice manifest obesity. Nevertheless, whether Crtc1 deficiency affects peripheral tissues during the development of obesity remains unclear. Peroxisome proliferator activated receptor γ (Pparγ) belongs to a family of nuclear receptors possessing transcriptional activity and regulating variety of genes (Surgucheva and Surguchov, 2008). It is a master regulator of whole-body lipid metabolism, adipogenesis, and insulin sensitivity (Ahmadian et al., 2013). Whether the obesity caused by Crtc1 deletion links to Pparγ remains unexplored.

In this study, we generated a Crtc1 knockout mouse line using the CRISPR/cas9 system, and investigated the impact of Crtc1 deletion on development and lipid metabolism. The molecular mechanism whereby Crtc1 regulating lipid metabolism in both liver and white adipose tissues was explored by RNA sequencing. Our findings shed a light on the lipogenesis during development and provide novel molecular targets to achieve weight loss.

## Materials and Methods

### Construction of Crtc1 Knockout Mouse

Crtc1 knockout (Crtc1^–/–^) mice were generated using CRISPR/Cas9 system. Exon 2 and 4 of Crtc1 gene were targeted by two single guide RNAs (gRNA). The gRNA sequences (5′∼3′) are GCTGACATCTGTGAATTGTA and CTGCATGCTGGATC GACAGG, respectively. We inter-crossed heterozygous females and heterozygous males for three generations to obtain the third generation homozygous Crtc1^–/–^ mice and littermate wild-type (Crtc1^+/+^) mice for formal experiments.

For Genotyping, genomic DNA from tail biopsies was obtained as previously described (Qi et al., 2006). Genotype identification primers (5′∼3′) were as follows: Crtc1-KO-F: CTTGCTGAGCCTCTTTGCCAG, Crtc1-KO-R: CCTTCAC ATCCTCCCAGAGATGTA; Crtc1-WT-F: GGCAACTGAGTT CATCCTAACACG; Crtc1-WT-R: CTTGTTCCCAAGAGGATC AAGGC. To determine Crtc1 knockout in various tissues of Crtc1^–/–^ mice at the mRNA levels, primers were designed for the missing exons, and the PCR products were identified by agarose gel electrophoresis. The primer sequences were as follows: Crtc1(Exon2-4)-F: TGCCCAACGTGAACCAGATT; Crtc1(Exon2-4)-R: CCCATGATGTCGTGTGGTCC.

### Animal Care

All experiments procedures were reviewed and approved by the Institutional Animal Care and Use Committee (IACUC) of Renmin Hospital of Wuhan University (No. 20180508) and performed in accordance with the guide for the care and use of laboratory animals published by National Institutes of Health, United States (8th edition). All mice were raised in a stable environment (room temperature, 24 ± 3°C; room humidity, 55 ± 5%) with a 12 h light/12 h dark cycle and fed normal chow. Six 5 months old Crtc1^–/–^ mice and six littermates Crtc1^+/+^ mice were randomly selected for food intake measurements. Mice were individually housed to avoid confounding factors related to social hierarchy within a cage. All mice were fed *ad libitum* for 2 weeks with food weight being measured daily between 5 and 6 p.m.

### Indirect Calorimetry

Four 5 months old Crtc1^–/–^ mice and four littermates Crtc1^+/+^ mice were randomly selected for indirect calorimetry measurement. Mice are individually housed within a cage in eight metabolic cages system (Beckman, CA, United States) and were adapted for 24 h before formal experiment, placed in a temperature-controlled space. In a sealed room, light was provided at 9:00 a.m.–9:00 p.m. which was regarded as daylight, and 9:01 p.m.–8:59 a.m. was thought to be dark. Oxygen consumption (VO_2_, ml/h/kg), carbon dioxide production (VCO_2_, ml/h/kg), RER (VO_2_/VCO_2_), spontaneous locomotor activities (counts) and heat production (kcal/kg/day) were monitored every 15 min for 24 h during the light and dark cycles.

### Intraperitoneal Glucose Tolerance Test (IPGTT)

After an overnight fasting (16 h), 50% glucose was injected intraperitoneally at a dose of 2 g/kg body weight to mice. The blood glucose of caudal vein at 0, 30, 60, 90, and 120 min after glucose loading was measured by a blood glucometer (Roche, Germany). The incremental area under the curve (AUC) for glucose was calculated using calculus method (mmol/L × h) by GraphPad Prism software v8.0 (United States).

### Sample Collection

Mice were anesthetized with inhalational anesthetic isoflurane for blood sampling from orbital sinus, and heparin-treated plasma were collected by centrifugation and stored at −80°C until use. Simultaneously, liver, eWAT (epididymal white adipose tissue), sWAT (subcutaneous white adipose tissue) and brown adipose tissues (BAT) were immediately dissected and weighted, a part of which were frozen in liquid nitrogen and stored in −80°C and the remaining were fixed in 4% paraformaldehyde.

### Western Blot

50 μg brain tissues were homogenized in 300 μl RIPA buffer containing protease and phosphatase inhibitor. After grinding 2 min, tissues were lysed for 30 min at 4°C. Total protein concentration was quantified using the BCA assay. Equivalent amounts of proteins were separated on 10% SDS-PAGE and transferred onto PVDF membranes. Membranes were blocked with 5% bovine serum albumin (BSA) for 1 h, and incubated overnight at 4°C with primary antibodies: CRTC1 (Cell Signaling Technology, United States, dilution: 1:1,000), GAPDH (Cell Signaling Technology, United States, dilution: 1:1,000), FLAG (Sigma, United States, dilution 1:1,000) and HA (Cell Signaling Technology, United States, dilution: 1:1,000). Then membranes were incubated with anti-rabbit secondary antibody for 2 h and detected by Odyssey system (LI-COR Biosciences).

### Plasma Biochemical Analyses

Plasma samples were measured for the levels of triglyceride (TG), total cholesterol (TCh) and fasting blood glucose (FBG) by Chemray 240/800 (Rayto) according to the manufacturer’s instructions. Fasting plasma fatty acid binding protein 4 (FABP4) and fasting insulin (FIN) levels were detected by Mouse FABP4 ELISA Kit (CSB-EL007945MO) and Mouse Insulin ELISA Kit (CSB-E05071m), respectively, in accordance with the manufacturer’s instructions. The absorbance was read at 450 nm. The homeostasis model assessment for insulin resistance (HOMA-IR) index was calculated as the formula: HOMA-IR = FBG ^∗^ FIN/22.5.

### Histopathology

Liver tissues and eWAT were fixed in 4% paraformaldehyde, embedded in paraffin, cut into 5 mm sections and stained with hematoxylin and eosin for H&E staining. For oil red O staining, the frozen liver sections were re-warmed and dried, fixed in the fixing solution for 15 min and stained with Oil Red O solution (Sigma Aldrich, St. Louis, MO, United States) for 8–10 min in the dark. Then they were soaked in 60% isopropanol for 3 s, and immediately counterstained with hematoxylin before drying. All slides were observed under the Olympus microscope (Model BX 71, Tokyo, Japan). Quantitative analysis of lipid-stained lesions was performed using ImageJ 5.0 software. To further determine the alterations of adipocyte size, three images of 100× magnification were chosen from each slide to calculate the mean area of adipocyte, which was performed by AdipoCount software (Shanghai, China).

### RNA Sequencing and Bioinformatics Analysis

Randomly selected two 12 weeks old male Crtc1^–/–^ mice and Crtc1^+/+^ mice were euthanatized by cervical dislocation after anesthesia with isoflurane. A piece of liver and eWAT were used for RNA extraction according to the manufacturer’s instructions. Transcriptome sequencing of RNA was completed by Beijing Genomics Institution (BGI). Two independent biological replicate samples were sequenced for each group. RNA-seq raw data and processed data have been uploaded to the GEO database (Accession code: GSE157270).

Normalization of expression matrix was accomplished by the normalize BetweenArrays function in R. The different expression genes (DEGs) between Crtc1^+/+^ and Crtc1^–/–^ samples were screened using linear models for microarray data (limma) package. | log2 (fold change) | > 1 and *P* < 0.01 was considered as the threshold. Gene ontology (GO) and Kyoto Encyclopedia of Genes and Genomes (KEGG) pathway enrichment analysis of DEGs were carried out by using DAVID online tools^1^. The integrated enrichment analysis of DEGs from liver and eWAT was implemented using Metasacape^2^ online database with default parameters. Gene Set Enrichment Analysis (GSEA) was used to screen significantly enriched signaling pathways with default parameters.

### Quantitative Real Time PCR (qRT-PCR)

Total RNA was extracted from the livers and eWAT of mice using Trizol reagent (TaKaRa, Shiga, Japan). The first strand cDNA was synthesized using RevertAid First Strand cDNA Synthesis Kit (Thermo Fisher Scientific, United States), and relative mRNA expression levels were analyzed by real-time qRT-PCR using LightCycler^®^ 480 (Roche, Switzerland). Sequences of primers were listed in Supplementary Table 2. 18S rRNA or β-actin were utilized to normalize the gene expression levels.

### Co-Immunoprecipitation (Co-IP)

Crtc1-Flag and Pparγ-HA plasmids were co-transfected into 293 T cells at ∼80% confluent. Cells were harvested and lysed for 30 min at 4°C in 500μl IP lysis buffer containing protease and phosphatase inhibitor. Supernatant was collected by centrifugation and pre-cleared by adding 20 μl protein G agarose beads (Thermo Fisher Scientific, IL, United States) at 4°C for 30 min. Then primary antibody was added (Flag, Sigma, United States, dilution:1:200; HA, Cell Signaling Technology, United States, dulition:1:200) and incubated overnight in 4°C rotation. Samples were incubated in 4°C rotation for 4 h with 20 μl protein G agarose beads washed by TBS the next day. Protein G agarose beads were collected after centrifuging at 5,000 rpm for 1 min, mixed with 100μl IP lysis buffer and analyzed by western blot, as described above. Input panels was used as the negative control.

### Statistical Analysis

Data was expressed as mean ± SEM. For normally distributed variables with equal variance, differences between two groups were evaluated using independent Student’s *t*-test. Multiple comparisons were analyzed by repeated measures ANOVA with Greenhouse-Geisser correction. P < 0.05 was considered statistically significant for two-tailed tests. Statistical analyses were performed by SPSS v22 (IBM; United States). Graphical visualization of transcriptome sequencing was realized by R software. The correlation analysis among Crtc1, Creb1 and Pparγ in adipose tissue was performed in Gene Expression Profiling Interactive Analysis (GEPIA^3^) with default conditions (Tang et al., 2017).

## Results

### Crtc1 Deficiency in Mice Causes Obesity and Infertility

**FIGURE 1:**
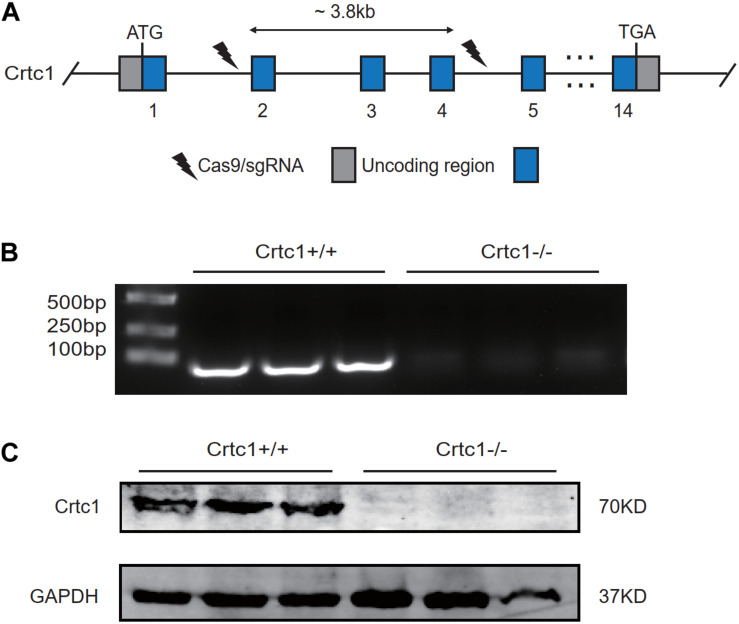
Construction of the Crtc1 knockout (Crtc1^–/–^) mouse. **(A)** Schematic diagram of knocking out Crtc1 gene using CRISPR/Cas9 system. **(B)** The mRNA level of Crtc1 was detected by agarose gel electrophoresis using Crtc1(Exon2-4)-F/R primers. **(C)** The protein level of Crtc1 was detected by Western blot.

To investigate the impact of Crtc1 in metabolism during development, we generated Crtc1^–/–^ mice using the CRISPR/Cas9 system (Figure 1A). Consistent with the report by Altarejos et al. (2008), but not with that by Breuillaud et al. (2009), we found that whole-body deficiency of Crtc1 caused severe impairment in fertility. Among three mating cages of Crtc1^–/–^ male and Crtc1^–/–^ female mice, only one pup was born and immediately died after birth. However, heterozygous Crtc1^+/–^ mice had normal fertility and gave birth to mice at the expected mendelian frequency (Table 1). DNA genotyping and (Figure 1B) and Western blot (Figure 1C) validated the complete knockout of Crtc1 in Crtc1^–/–^ mice.

**TABLE 1:** Information on offspring mice of heterozygous Crtc1^+/–^ mice mating.

Genotype	Mendelian expected	Actual number (male/female)
Crtc1^+/+^	20	22 (10/12)
Crtc1^+/–^	40	35 (13/22)
Crtc1^–/–^	20	23 (14/9)
Total	80	80

**FIGURE 2:**
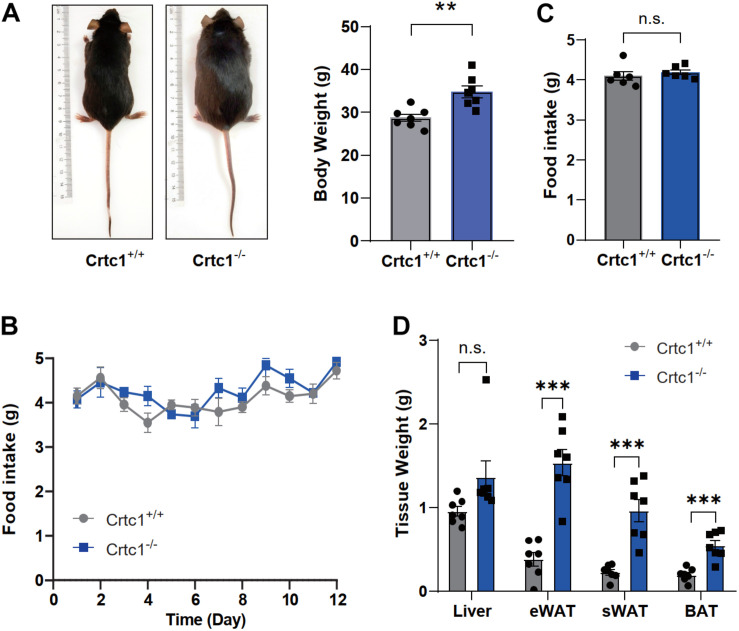
Crtc1 deficiency induces obesity in mice. **(A)** Representative images of appearance of Crtc1^+/+^ and Crtc1^–/–^ mice. **(B)** Food intake of Crtc1^+/+^ and Crtc1^–/–^ mice during pair feeding. **(C)** Average body weight and daily food intake in the two groups. **(D)** Effect of Crtc1 deficiency on the weight of Liver, eWAT, sWAT and BAT. Data are mean ± SEM; **P < 0.01, ***P < 0.001 and n.s. indicates no significant difference.

Under normal feeding conditions, Crtc1^–/–^ mice were larger and heavier than Crtc1^+/+^ mice at the age of 8 months (Figures 2A,B). This increase was obviously contributed by the increased weights of fats including eWAT, sWAT and BAT (Figure 2D). In contrast to a previous report (Altarejos et al., 2008), there was no difference in daily food intake and averaged food intake between Crtc1^–/–^ and Crtc1^+/+^ groups (Figures 2B,C).

**FIGURE 3:**
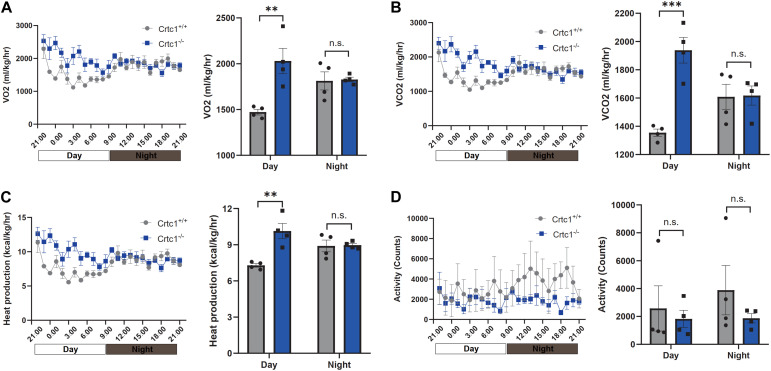
Effect of Crtc1 on indirect calorimetry. **(A)** O_2_ consumption curve and average O_2_ consumption. **(B)** CO_2_ production curve and average CO_2_ production. **(C)** Heat production curve and average heat production. **(D)** Activities counts curve and activities counts, measured over 24 h in Crtc1^+/+^ and Crtc1^–/–^ mice at the age of 6 months. Data are mean ± SEM; **P < 0.01, ***P < 0.001 and n.s. indicates no significant difference.

We then performed indirect calorimetry measurement using metabolic cages to investigate the impact of Crtc1 deficiency on over-all energy metabolism. Compared with Crct1^+/+^ mice, Crtc1^–/–^ mice had higher oxygen consumption (Figure 3A), carbon dioxide production (Figure 3B) and heat production (Figure 3C) in day-time (inactive phase), but there was no significant difference of the above parameters in night-time (active phase) between the two groups. Though not reaching significance due to large variations, physical activity of Crtc1^–/–^ mice during night-time was generally lower than that of Crtc1^+/+^ mice (Figure 3D). Interestingly, energy metabolism of Crct1^+/+^ mice conformed to normal circadian rhythm, which could not be observed in Crtc1^–/–^ mice (Figures 3A–D). These data suggest that obesity in Crtc1^–/–^ mice seems not to be the result from abnormal food intake nor from reduced energy expenditure. However, these mice show alterations in lipid metabolism in adipose tissue which might be the reason for obesity development in these mice.

### Crtc1 Deficiency Causes Metabolic Disorders

**FIGURE 4:**
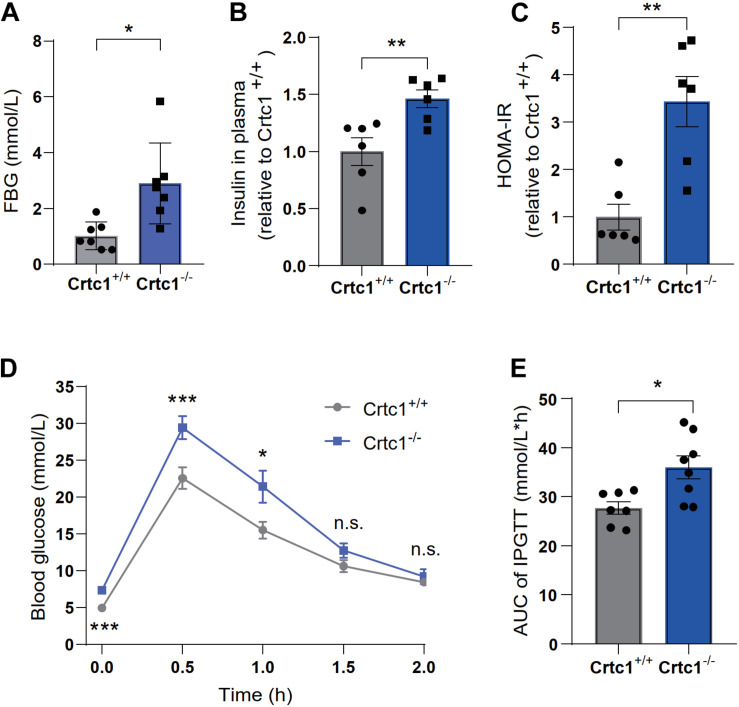
Crtc1 deficiency damages glucose tolerance in mice. **(A)** Fasting blood glucose (FBG). **(B)** Fasting plasma insulin, **(C)** and HOMA-IR. **(D)** The curve of blood glucose level with time. **(E)** Area under the plasma glucose concentration curve (AUC) in Crtc1^+/+^ and Crtc1^–/–^ mice at the age of 8 months. Data are mean ± SEM; *P < 0.05; **P < 0.01, ***P < 0.001 and n.s. indicates no significant difference.

Compared to Crtc1^+/+^ mice, Crtc1^–/–^ mice exhibited lower insulin sensitivity, as demonstrated by elevated fasting blood glucose (Figure 4A), fasting plasma insulin (Figure 4B), and HOMA-IR (Figure 4C). The results of IPTGG further confirmed that Crtc1 deficiency impaired glucose tolerance in mice (Figures 4D,E).

**FIGURE 5:**
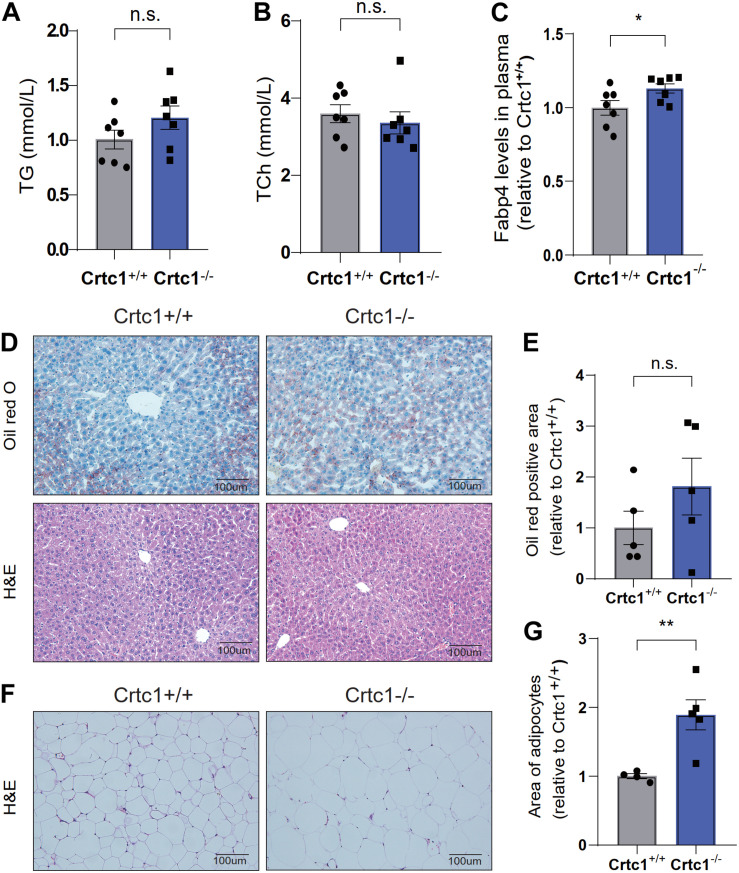
Abnormal lipid metabolism in Crtc1^–/–^mice. **(A)** Plasma triglyceride (TG). **(B)** Plasma total cholesterol (TC). **(C)** Plasma fatty acid binding protein 4 (FABP4) in Crtc1^+/+^ and Crtc1^–/–^ mice at the age of 8 months. **(D)** Oil Red O and H&E staining of the liver tissues from Crtc1^+/+^ and Crtc1^–/–^. **(E)** Quantification of the Oil Red positive area of liver. **(F)** H&E staining of white adipose tissue from Crtc1^+/+^ and Crtc1^–/–^ mice. **(G)** Quantification of the mean area for adipocyte. Data are mean ± SEM; *P < 0.05; **P < 0.01 and n.s. indicates no significant difference.

Although there was no statistical difference in plasma TC and TG between the two groups, Crtc1^–/–^ mice had higher levels of plasma FABP4, an adipokine mainly secreted by adipocytes (Figures 5A–C). Liver and adipose are the major tissues governing lipid metabolism. H&E and Oil Red O staining showed marginal effects of Crtc1 deficiency on liver morphology and lipid droplets (Figures 5D,E). However, we found that the mean area of adipocytes from Crtc1^–/–^ eWAT significantly increased compared with Crtc1^+/+^ mice (Figures 5F,G). These data implicate that the increased insulin resistance after Crtc1 deletion might be attributable to the fat accumulation in adipose tissue.

### Crtc1 Deficiency Globally Modifies Lipid Metabolism in Adipose Tissues

**FIGURE 6:**
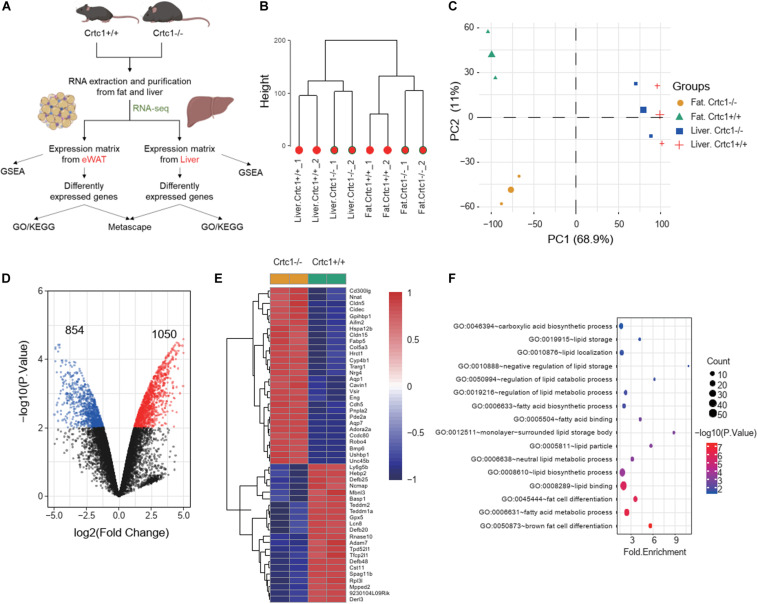
Crtc1 deficiency globally remodels lipid metabolism in adipose tissue. **(A)** Schematic of the transcriptome analysis procedure. **(B)** Hierarchical clustering showing the relationship among different groups at the transcriptome level. **(C)** PCA plot showing global sample distribution profiles. **(D)** Volcano plot of DEGs (red, upregulated genes; blue, downregulated genes) in eWAT under the cut-off value of | log2 (fold change) | > 1 and *P* < 0.01. **(E)** Heatmap of the top 50 most significant DEGs in eWAT. **(F)** Bubble plot of lipid-metabolism-related biological processes (*P* < 0.05) in eWAT by GO enrichment analysis.

To investigate the underlying mechanism, we performed transcriptome analyses using liver and eWAT tissues from Crtc1^+/+^ and Crtc1^–/–^ mice (Figure 6A). Clustering tree and the principal component analysis (PCA) illustrated that Crtc1 deletion had a greater impact on the transcriptome in eWAT than that in liver (Figures 6B,C). Volcano plots showed that there were more DEGs between Crtc1^–/–^ and Crtc1^+/+^ in eWAT (1,050 upregulated genes and 854 downregulated genes) than liver (386 upregulated genes and 97 downregulated genes) when the cut-off value of | log2 (fold change) | was > 1 and *P* < 0.01 (Figure 6D and Supplementary Figure 1A). The top 50 DEGs in eWAT (Figure 6E) and liver (Supplementary Figure 1B) were shown as heatmaps.

The results of GSEA (Table 2) and GO enrichment analysis (Figure 6F) of DEGs in eWAT showed that biological processes affected by Crtc1 deletion were closely related to fatty acid metabolism and adipocyte differentiation. These processes included brown fat cell differentiation, fatty acid metabolic process, fat cell differentiation, lipid binding and lipid biosynthetic process in GO analysis (Figure 6F), and fatty acid beta oxidation, fatty acid catabolic process, lipid oxidation, monocarboxylic acid catabolic process, regulation of fatty acid oxidation and fatty acid metabolic process in GSEA (Table 2).

**TABLE 2:** Top 10 GO term significantly enriched in GSEA of Fat.

Term	Size	ES	NES	*P*-value	FDR
Fatty acid beta oxidation	67	0.6354	2.817212	0	0
Respiratory chain complex	66	0.604227	2.748158	0	0
Fatty acid catabolic process	91	0.585858	2.734791	0	0
Lipid oxidation	90	0.56813	2.632225	0	0
Monocarboxylic acid catabolic process	105	0.539191	2.606093	0	0
Regulation of fatty acid oxidation	26	0.72454	2.539472	0	0
Organic acid catabolic process	207	0.465007	2.519613	0	0
Fatty acid metabolic process	188	0.478127	2.512534	0	2.11E–04
Inner mitochondrial membrane protein complex	96	0.521155	2.465609	0	1.88E–04
Cellular response to vascular endothelial growth factor stimulus	43	0.610762	2.457331	0	1.69E–04

In contrast, DEGs in liver were not relevant to lipid metabolism, but were significantly enriched in extracellular region, extracellular matrix, regulation of locomotion, extracellular matrix part and proteinaceous extracellular matrix (Supplementary Figure 1C). This result was further confirmed by GSEA in liver (Supplementary Table 1). In addition, integrated enrichment analysis of DEGs between liver and eWAT also showed that the fat accumulation caused by Crtc1 deletion was largely related to adipose but not liver (Supplementary Figure 1E).

**FIGURE 7:**
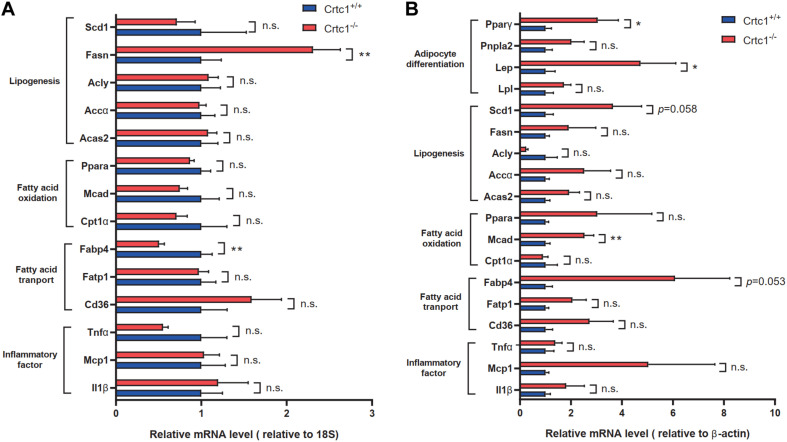
Validation of lipid-metabolism-related genes. **(A,B)** Expression of key genes related to different lipid metabolism processes in liver **(A)** and eWAT **(B)**, measured by qRT-PCR. Data are mean ± SEM; *P < 0.05; **P < 0.01 and n.s. indicates no significant difference between Crtc1^+/+^ and Crtc1^–/–^ groups. *n* = 6.

To validate these findings, we examined the mRNA levels of lipid-metabolism-related genes by qRT-PCR. In liver, there was no significant difference in the expression of key genes related to lipogenesis, fatty acid transport and fatty acid oxidation, except for Fasn and Cd36, between Crtc1^–/–^ and Crtc1^+/+^ mice (Figure 7A). However, the mRNA levels of genes related to fat differentiation, lipogenesis, fatty acid transport oxidation and transport were substantially increased in adipose tissue of Crtc1^–/–^ mice compared with Crtc1^+/+^ mice (Figure 7B).

### Crtc1 Deficiency Promotes Lipogenesis Potentially via Activating PPARγ Signaling Pathway

**FIGURE 8:**
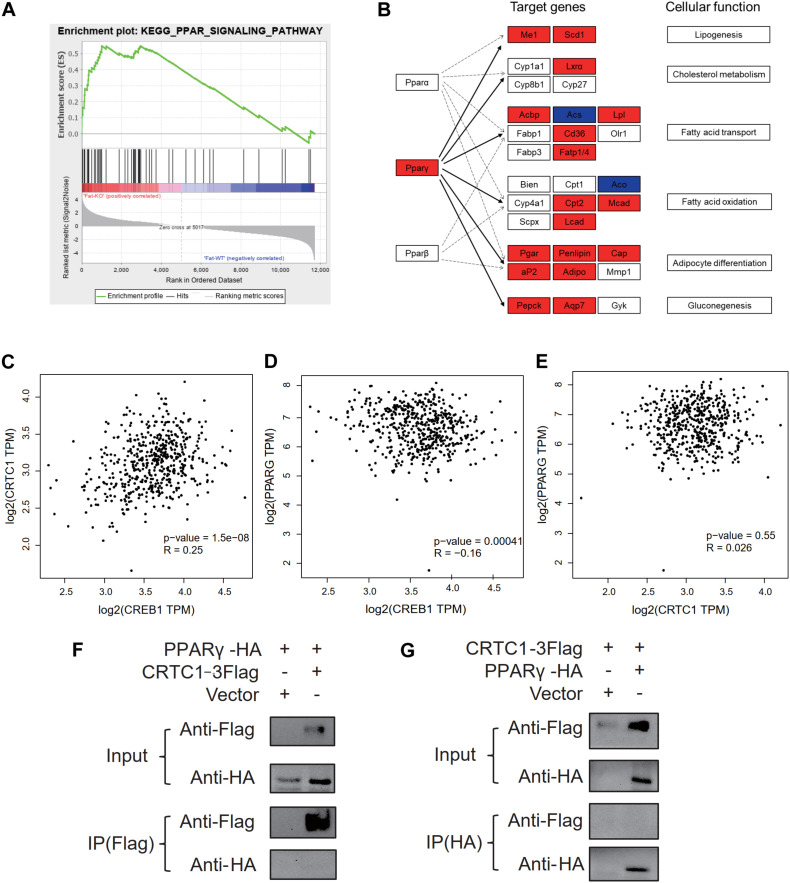
Crtc1 deletion up-regulated the PPAR signaling pathway. **(A)** PPAR signaling pathway was enriched in GSEA. **(B)** Enrichment analysis of DEGs in eWAT tissue in PPAR pathway using KEGG map. Red represents upregulated genes. Blue represents downregulated genes. **(C–E)** Scattered plot of correlation between Crtc1, Creb1 and Pparγ in human adipose tissue using GEPIA. **(F,G)** Co-IP validation of protein interactions between CRTC1 and PPARγ by Flag primary antibody(F) and HA primary antibody **(G)**, respectively.

According to the results of GSEA (Figure 8A) analysis and KEGG (Figure 8B) analysis, we found that PPAR signaling pathway was significantly up-regulated in Crtc1^–/–^ mice. More importantly, transcription factor Pparγ was significantly enriched in DEGs of eWAT by Metascape enrichment analysis (Table 3). Activation of Pparγ in white adipocytes has been shown to promote fatty acid storage, triglyceride (TG) synthesis and glucose uptake through upregulation of fatty acid metabolism genes, including C/EBPα, Stat1, Stat5, Fabp4, Lpl, Cd36, Glut4, Pepck, and etc. (Ahmadian et al., 2013; Marechal et al., 2018). Most of these genes were remarkably up-regulated in eWAT in Crtc1^–/–^ mice (Figure 8B). These results suggest that Crtc1 deficiency promotes lipogenesis potentially via activating PPARγ signaling pathway.

**TABLE 3:** Top 10 transcription factor enriched in Metascape of eWAT.

Transcription factor	−log10 (*P*-value)	Enrichment	*Z*-score	−log10 (*Q*-value)
Sp1	6.3	2.1	5.6	5.6
PPARg	6.2	3.8	6.3	6.3
PPARa	6	3.9	6.2	6.2
Nr3c2	4	8	5.8	5.8
Clock	3.8	5.3	5.1	5.1
PPARd	3.8	6.1	5.3	5.3
Thrap3	3.2	11	5.5	5.5
Cebpb	3.1	2.9	3.9	3.9
Egr1	2.9	2.4	3.6	3.6

In addition to the transcriptional regulation of Pparγ expression in adipose tissue (Figure 7B), we also explored whether CRTC1, as a transcription cofactor, might directly bind to PPARγ to modify its activity. However, the Co-IP assay failed to observe a direct interaction between CRTC1 and PPARγ in 293T cell (Figures 8F,G). Interestingly, from the online databases of healthy population, we found that Crtc1 was positively correlated with Creb1 (*R* = 0.25, *P* < 0.001) but not with Pparγ (*R* = 0.026, *P* = 0.55) at the mRNA level, whereas Creb1 was negatively correlated with Pparγ (*R* = −0.16, *P* < 0.001) (Figures 8C–E). These data imply that Crtc1 might regulate Pparγ transcription via Creb1.

## Discussion

Obesity is characterized by excessive fat accumulation and associated with glucose and lipid metabolism disorders. Crtc1, a transcription cofactor regulating CREB activity, has been involved in the pathogenesis of metabolic syndrome. However, the effect of Crtc1 on metabolism in peripheral tissues and the underlying mechanisms remain unclear. In our study, we found that Crtc1 deficiency aggravated the progression of fat accumulation potentially through activating PPARγ signaling pathway.

CRTCs family members have been closely associated with lipid metabolism. Crtc2 has been implicated as a critical mediator of increased Srebp1 activation and enhancement of de novo lipogenesis, which consequently controls hepatic lipid levels (Han et al., 2015). In addition, Song et al. (2010) has discovered that Crtc3 induces obesity by weakening β-adrenergic receptor in adipose, and Crtc3^–/–^ mice are protected from hepatic steatosis under high-fat-diet feeding conditions. Given that Crtc2 and 3 show 32% homology with Crtc1 (Iourgenko et al., 2003), we speculate that Crtc1 has greater potential for modulating whole-body lipid homeostasis.

In this study, we firstly identified transcription cofactor Crtc1 as one of genes to generate anti-obesity effect, reflected by increase of bodyweight and fat weight including sWAT, eWAT and BAT in Crtc1^–/–^ mice. We performed food intake and indirect calorimetry measurements to determine the reason that Crtc1^–/–^ mice were heavier than Crtc1^+/+^ mice. The results excluded the possibility that abnormal food intake or reduced energy expenditure is the main contribution to obesity in Crtc1^–/–^ mice. It is inferred that Crtc1 maintains the whole-body energy balance, and Crtc1^–/–^ mice are predisposed to synthesize fat, not mobilize fat. Another possible contributor to obesity may be derived from the impact of Crtc1 on food absorption, which needs validation in future studies. Interestingly, Crtc1 deletion also impairs normal circadian rhythm on energy metabolism, which confirms a previous report that CRTC1-SIK1 pathway regulates the entrainment of the circadian clock (Jagannath et al., 2013). The crosstalk of circadian rhythm and lipid metabolism is increasingly recognized, and this interaction may be involved in the maintenance of metabolism homeostasis. Cumulating clinical and animal studies emphasize that the disruption of circadian rhythm widely affects lipid metabolism and accelerates the occurrence and development of obesity (Albrecht, 2017), which may be the crucial reason for the obesity in Crtc1^–/–^ mice. Importantly, Crtc1^–/–^ mice displayed insulin resistance but normal lipid levels compared to Crtc1^+/+^ mice at 8 months old, indicating that insulin sensitivity is earlier than plasma lipid profiles to sense fat accumulation. Aforementioned results suggest a possible role of Crtc1 in regulating lipids delivery and accumulation in peripheral tissues.

In addition to white adipose tissue, liver is the pivotal peripheral organ to maintain lipid homeostasis. Kim (2016) previously reported that Crtc1^–/–^ mice developed spontaneous hepatic steatosis and systemic deterioration in lipid, cholesterol, and bile acid homeostasis became more apparent with age. However, in our study, both H&E and oil red O staining assays showed no significant difference in liver steatosis induced by Crtc1 knockout at the age of 8 months. Although RNA sequencing analysis showed that the transcriptome alteration in the liver caused by Crtc1 deletion was not significantly associated with lipid metabolism, PCR validation assays found that the expression of Fasn and Cd36 which are related to the synthesis of fatty acid were upregulated in Crtc1^–/–^ mice. Therefore, alterations in liver metabolism might still be present, and its contribution to the obesity in Crtc1-deficient mouse need to be further investigated in future.

The area of adipocytes, in particularly eWAT, showed a uniform and significant increase in Crtc1^–/–^ mice confirmed by H&E staining, suggesting that the impact of Crtc1 deficiency on obesity was specific to white adipose tissues. Pathological white adipose remodeling, typically characterized by adipocyte hypertrophy, chronic inflammation and fibrosis, is associated with risks of metabolic diseases (Vishvanath and Gupta, 2019). It is well known that adipocyte hypertrophy and increased adiponectin secretion in the adipose tissue are responsible for insulin resistance (Lu et al., 2020), consistent with the increased circulating FABP4 and insulin levels in Crtc1^–/–^ mice observed in our study. Moreover, clinical evidence also indicates that Crtc1 polymorphisms increase body mass index and fat mass in the general adult population (Choong et al., 2013), and Crtc1 DNA methylation is related to fat distribution (Rohde et al., 2019). Transcriptome analysis of eWAT showed that the biological processes affected by Crtc1 deletion were closely related to fatty acid metabolism and adipocyte differentiation. The results of qRT-PCR further validated that Crtc1 deletion resulted in increased expression of genes related to adipocyte differentiation (Pparγ, Lep) and fatty acid transportation (Fabp4), which convergently caused fat accumulation in eWAT with normal chow diet. Collectively, these findings suggest that the regulation of lipid metabolism by Crtc1 might happen in adipose tissue, but not in liver.

According to our RNA-seq data, PPAR signaling pathway and transcription factor Pparγ were significantly enriched in KEGG analysis and Metascape of DEGs in eWAT, respectively. In addition, PPAR signaling pathway was also enriched using GSEA analysis by functional class scoring method. The results of the comprehensive analysis consistently showed that Pparγ played an important role in the lipid accumulation in adipose tissue caused by Crtc1^–/–^. Pparγ is thought to be a master transcription factor of adipogenesis (Lefterova et al., 2014). Once adipogenesis program being initiated, a transcriptional cascade is activated and induces the expression of metabolic genes and adipokines associated with the adipocyte phenotype, such as Fabp4, Glut4, leptin and adiponectin (Lefterova and Lazar, 2009).

CRTCs are inactivated through phosphorylation by AMP-activated protein kinase (AMPK) family kinases, such as salt-inducible kinases 1, 2, and 3 (SIK1/2/3), and inversely they are activated via dephosphorylation by the serine/threonine phosphatase calcineurin, which induces nuclear translocation and consequently activates CREB targets (Escoubas et al., 2017). Creb1 is an important transcription factor regulating Pgc1α content, a transcriptional coactivator interacting with Pparγ (Singh et al., 2015). It has been reported that Malat1, a long non-coding RNA, maintains the phosphorylation of CREB to further activate the CREB signaling pathway, thus enhancing PGC-1α level and PPARγ activity (Ruan et al., 2019). However, Herzig et al. (2003) found that Creb1^–/–^ inhibited the transcription activity of Hes1, which was a transcription repressor of Pparγ, then upregulated the level of Pparγ. Moreover, Creb1 has been shown to aggravate insulin resistance by stimulating the expression of the bZiP factor ATF3 which represses Pparγ expression and inhibits adipocyte differentiation (Qi et al., 2009; Jang and Jung, 2014). In NIH 3T3 cells, Crtc1 acts as a sensor of stimulation, triggering CREB-driven transcription of SIK1 and PER1 and thereby regulating circadian rhythms (Jagannath et al., 2013). Similarly, CREB-CRTC1 pathway participants in energy balance and long-term hippocampal plasticity in this manner (Altarejos et al., 2008; Ch’ng et al., 2012). We further analyzed the online database of health population and found that Creb1 negatively regulated the mRNA levels of Pparγ but there was no correlation between Crtc1 and Pparγ at the mRNA levels. Therefore, the up-regulation of Pparγ expression by Crtc1 deficiency might be attributable to the suppression of CREB1 activity, a potential molecular link that needs further investigation.

The absence of Crtc1 strikingly impaired the reproduction in mice even if not causing complete infertility. This observation is different from previous reports (Altarejos et al., 2008; Breuillaud et al., 2009), where the conclusions were controversial regarding the effect of Crtc1 deletion on fertility. Altarejos (Altarejos et al., 2008) reported that Crtc1^–/–^ females had abnormal uterine morphology and low circulating luteinizing hormone levels via down-regulation of Kiss1 gene expression. However, although the same gene trap method was used to construct Crtc1 knockout mice, the results from Breuillaud’s study were completely opposite (Breuillaud et al., 2009). Breuillaud et al. speculated that the reason for the opposite conclusion might be the different background of C57BL/6N mice, or the different gene background generated in the process of constructing Crtc1 knockout mice by gene trap. Our results supported that the lack of Crtc1 impaired fertility by a different knockout strategy using the CRISPR/Cas9 system. Obesity is associated with various reproductive sequelae in females, including anovulation, subfertility, infertility and increased risk of miscarriage due to dysregulation of the hypothalamic-pituitary-ovarian axis (Broughton and Moley, 2017). Similarly, obesity also causes male-factor infertility by means of endocrine abnormalities, and directly effects on the fidelity and throughput of spermatogenesis (Craig et al., 2017). Whether the impaired fertility of Crtc1^–/–^ mice is a result of obesity *per se* needs to be investigated in future.

In summary, our data demonstrate that the obesity caused by Crtc1^–/–^ deficiency was associated with increased fat accumulation in white adipose tissues, which potentially via activating PPARγ signaling pathway. These findings provide novel insights into the treatment of obesity.

## Data Availability Statement

The datasets presented in this study can be found in online repositories. The names of the repository/repositories and accession number(s) can be found in the article/Supplementary Material.

## Ethics Statement

The animal study was reviewed and approved by the Institutional Animal Care and Use Committee of Renmin Hospital of Wuhan University (application number 20180508).

## Author Contributions

ZW and MF conceived and designed the project. YHu, JL, and YF performed the experiments with input from YHe and LL. JL performed the bioinformatic analyses of RNA-seq data. YHu and JL drafted the manuscript. All authors have read and consent to the content of the manuscript.

## Conflict of Interest

The authors declare that the research was conducted in the absence of any commercial or financial relationships that could be construed as a potential conflict of interest.

## Footnotes

1 http://david.ncifcrf.gov

2 https://metascape.org/gp/index.html

3 http://gepia.cancer-pku.cn/index.html
