# Supplementary material for: Crtc1 Deficiency Causes Obesity Potentially via Regulating PPARγ Pathway in White Adipose
Source: Front Cell Dev Biol. 2021 Apr 12;9:602529. doi: 10.3389/fcell.2021.602529 (PMC8075410; doi:10.3389/fcell.2021.602529)
Supplement: Supplementary file 1 [file Table_1.DOCX]

Supplementary Material

# Supplementary Tables

Supplement Table 1. Top 10 GO term significantly enriched in GSEA of Liver.

| Term | Size | ES | NES | P value | FDR |
| --- | --- | --- | --- | --- | --- |
| Cotranslational protein targeting to membrane | 93 | 0.615062 | 2.434873 | 0 | 0 |
| Establishment of protein localization to endoplasmic reticulum | 103 | 0.570264 | 2.279016 | 0 | 0 |
| Nuclear transcribed mrna catabolic process nonsense mediated decay | 115 | 0.531766 | 2.213309 | 0 | 0.001228 |
| Cytosolic ribosome | 99 | 0.549616 | 2.193877 | 0 | 0.001379 |
| Protein localization to endoplasmic reticulum | 125 | 0.524965 | 2.183407 | 0 | 0.001287 |
| Cytosolic large ribosomal subunit | 54 | 0.579823 | 2.091313 | 0 | 0.007505 |
| Polysomal ribosome | 28 | 0.651076 | 2.083055 | 0 | 0.007485 |
| Glycosaminoglycan binding | 124 | 0.502225 | 2.081905 | 0 | 0.00655 |
| Cytokine activity | 60 | 0.552876 | 2.036005 | 0 | 0.014397 |
| Mitochondrial electron transport nadh to ubiquinone | 44 | 0.573036 | 2.023503 | 0 | 0.016549 |

Supplement Table 2. qRT-PCR primers for mice.

| Gene Symbol | Forward primer | Reverse primer |
| --- | --- | --- |
| Tnf-α | CATCTTCTCAAAATTCGAGTGACAA | TGGGAGTAGACAAGGTACAACCC |
| Il-1β | CCGTGGACCTTCCAGGATGA | GGGAACGTCACACACCAGCA |
| Mcp1 | TACAAGAGGATCACCAGCAGC | ACCTTAGGGCAGATGCAGTT |
| Fabp4 | CCGCAGACGACAGGA | CTCATGCCCTTTCATAAACT |
| Fatp1 | TGCACAGCAGGTACTACCGCAT | TGCGCAGTACCACCGTCAAC |
| Cd36 | GACTGGGACCATTGGTGATGA | AAGGCCATCTCTACCATGCC |
| Cpt1α | AGGACCCTGAGGCATCTATT | ATGACCTCCTGGCATTCTCC |
| Pparα | TATTCGGCTGAAGCTGGTGTAC | CTGGCATTTGTTCCGGTTCT |
| Mcad | GAAGCCACGAAGTATGCCCT | TAGTAAGTGTTCCGGCGACC |
| Accα | GGCCAGTGCTATGCTGAGAT | AGGGTCAAGTGCTGCTCCA |
| Fasn | CTGCGGAAACTTCAGGAAATG | GGTTCGGAATGCTATCCAGG |
| Scd1 | TCTTCCTTATCATTGCCAACACCA | GCGTTGAGCACCAGAGTGTATCG |
| Acas2 | GCTGAACTGACACACCTGGA | AACTTGGCGACAAAGTTGCT |
| Acly | AATGGCCGTCATGTGAGTTT | GTGGCCCCAACTATCAAGAG |
| Lep | GTGGCTTTGGTCCTATCTGTC | CGTGTGTGAAATGTCATTGATCC |
| Pnpla2 | ATGTTCCCGAGGGAGACCAA | GAGGCTCCGTAGATGTGAGTG |
| Lpl | TTGCCCTAAGGACCCCTGAA | TTGAAGTGGCAGTTAGACACAG |
| Pparγ | ATTCTGGCCCACCAACTTCGG | TGGAAGCCTGATGCTTTATCCCCA |
| β-actin | GTGACGTTGACATCCGTAAAGA | GCCGGACTCATCGTACTCC |
| 18S | AGGGTTCGATTCCGGAGAGG | CAACTTTAATATACGCTATTGG |

# Supplementary Figures


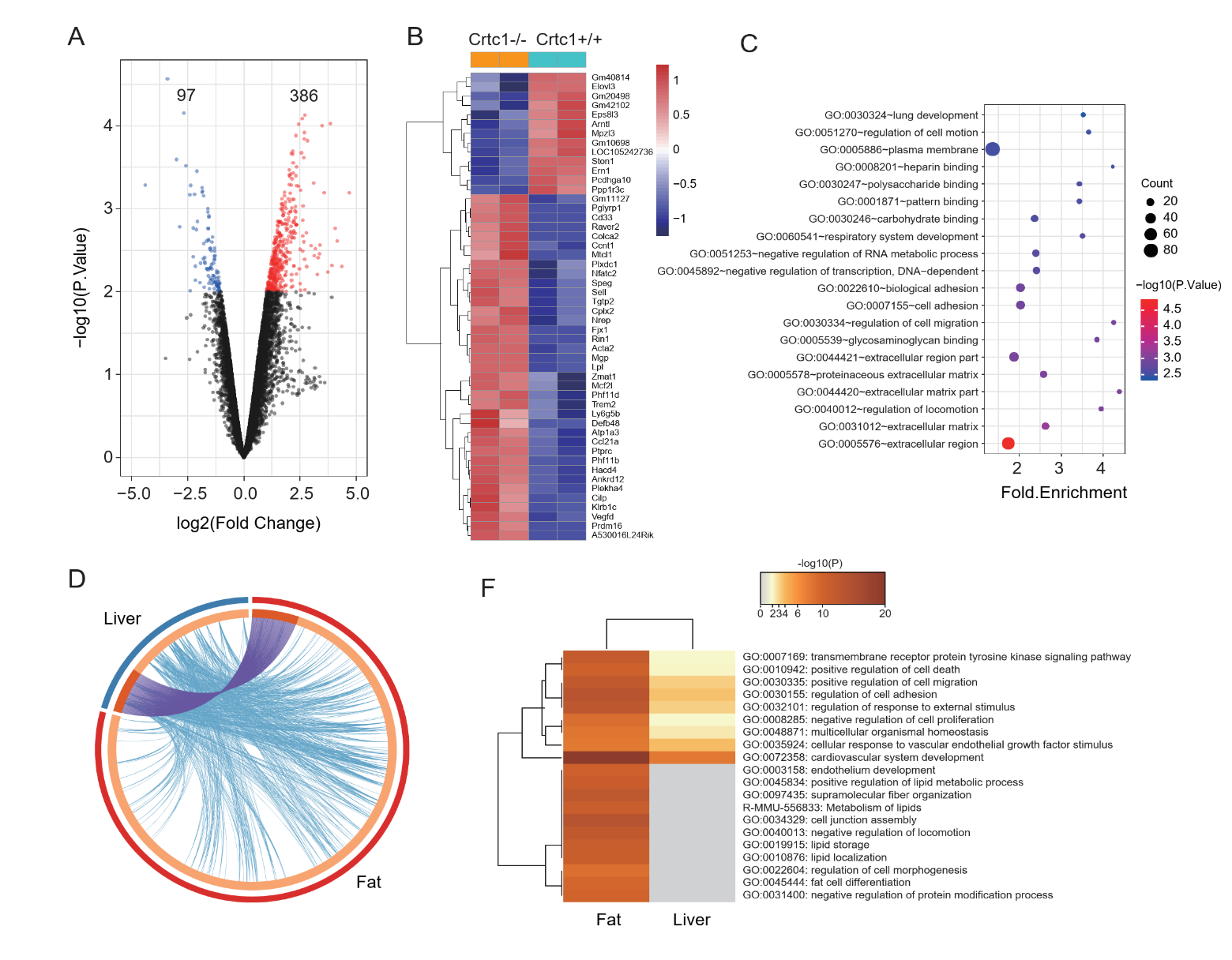


Supplement Figure 1. Liver transcriptome analysis between Crtc1^-/-^ and Crtc1^+/+^ mice. (A) Volcano plot of DEGs (red, upregulated genes; blue, downregulated genes) in liver under the cut-off value of |log2 (fold change) | >1 and P-value < 0.01. (B) Heatmap of the top 50 most significant DEGs in liver. (C) Bobble plot of top 20 enriched GO terms in liver by GO analysis. (D) Overlap of DEGs in eWAT and liver using Metascape. The inner circle represents DEGs in eWAT or liver. Purple curves link identical genes between eWAT and liver, where blue curves link genes that belong to the same enriched ontology term. Genes that hit multiple lists are colored in dark orange, and genes unique to a list are shown in light orange. (E) Heatmap of enriched terms between eWAT and liver using Metascape. Colored by P-value.
